# Supplementary figures and images for: Shp2 in uterine stromal cells critically regulates on time embryo implantation and stromal decidualization by multiple pathways during early pregnancy
Source: PLoS Genet. 2022 Jan 13;18(1):e1010018. doi: 10.1371/journal.pgen.1010018 (PMC8791483; doi:10.1371/journal.pgen.1010018)

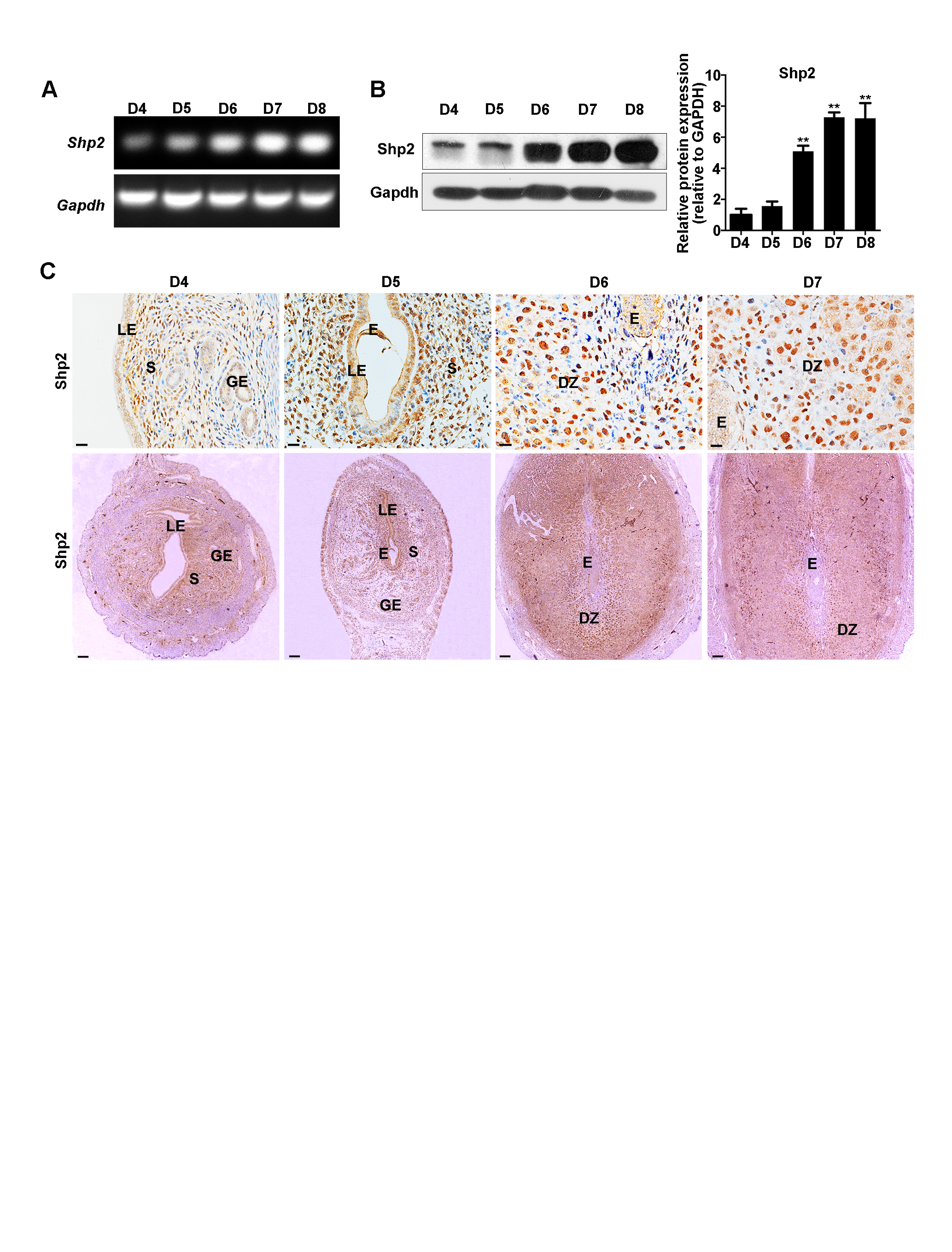

Supplement: S1 Fig — (A) Reverse transcription PCR analysis of wild-type uterine Shp2 mRNA levels on D4-D8 of pregnancy (n = 6). (B) Western blot detection of Shp2 protein levels in wild-type uteri from D4-D8. (C) Immunohistochemical staining of Shp2 in local area (up) and full tissues (down) of wild-type D4-D7 uteri (n = 6). Scale bars for the top 4 images in panel C, 20 μm; Scale bars for the bottom four images in panel C, 200 μm. The data are presented as the mean ± SD from at least 6 mice in each group or three independent experiments. Statistical differences are indicated as follows: *P<0.05, **P<0.01. (TIF) [file pgen.1010018.s001.tif]

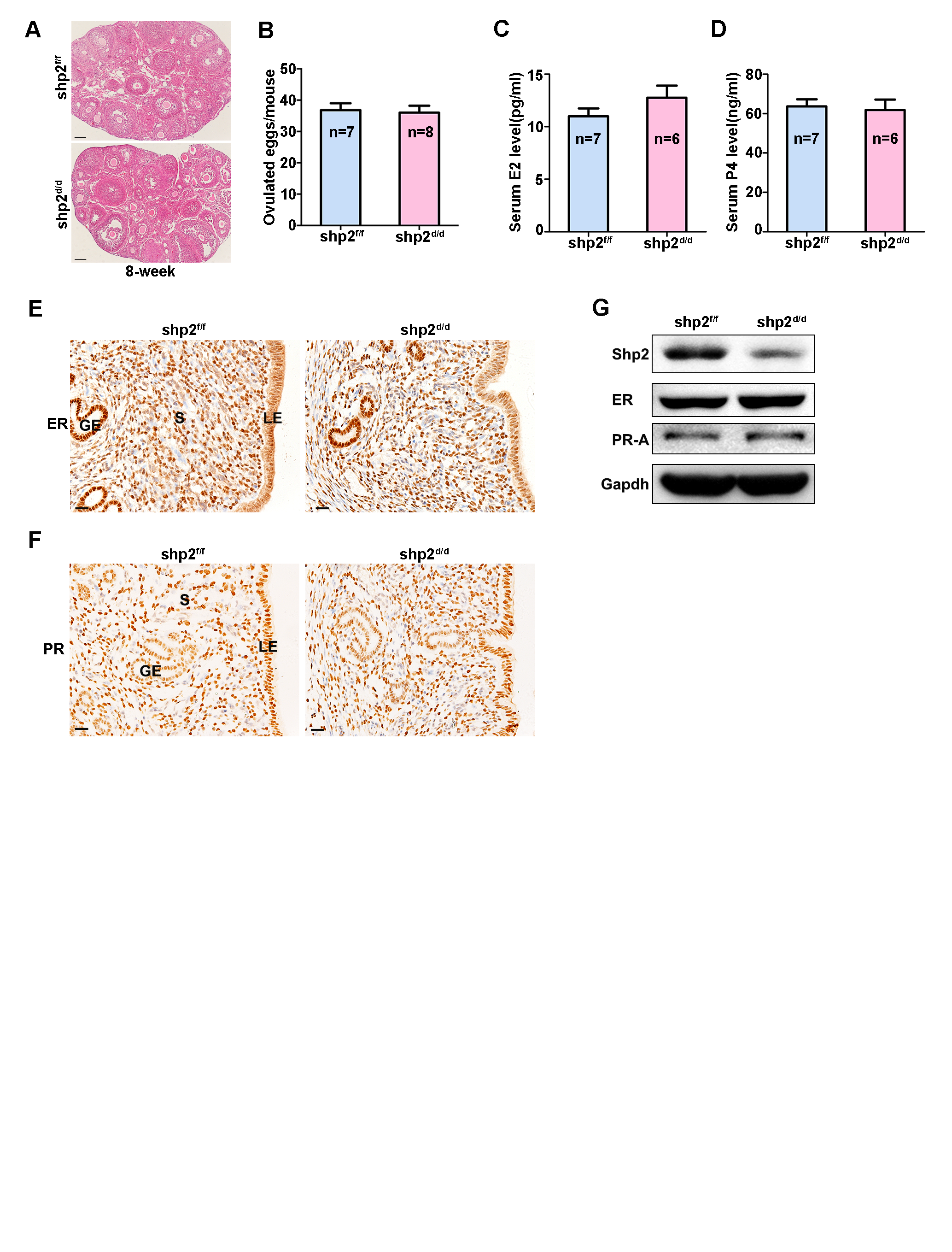

Supplement: S2 Fig — (A) Hematoxylin and eosin staining showed similar ovarian histology between Shp2f/f (n = 7) and Shp2d/d (n = 8). Scale bars, 100μm. (B) In superovulation assay, Shp2d/d (n = 8) mice ovulated comparable oocytes to that of the control mice (n = 7) at 16 h after hCG. (C-D) Serum levels of E2 and P4 in Shp2f/f (n = 7) and Shp2d/d mice (n = 6) on D4 of pregnancy. Number within the bar indicated the number of mice tested. (E-F) Immunohistochemistry staining of ER (E) and PR (F) in Shp2f/f (n = 7) and Shp2d/d (n = 6) uteri on day 4 of pregnancy. (G) Western blotting of ER and PR in Shp2f/f and Shp2d/d uteri on day 4 of pregnancy. Gapdh serves as loading control. The data are presented as the mean ± SD from at least 6 mice in each group or three independent experiments. Statistical differences are indicated as follows: *P<0.05, **P<0.01. (TIF) [file pgen.1010018.s002.tif]

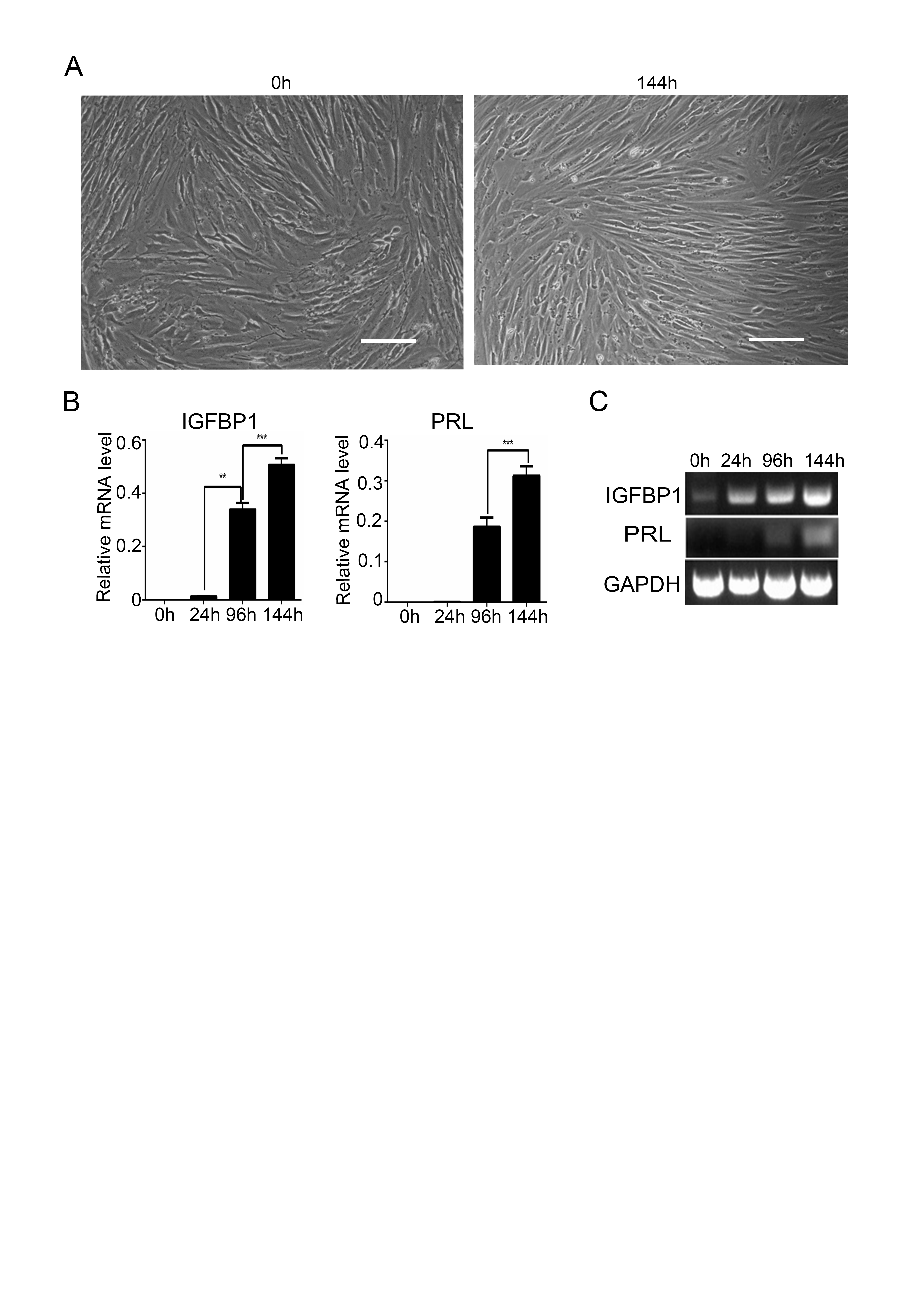

Supplement: S3 Fig — (A) The left picture shows the bright field picture of the undifferentiated uterine stromal cells, and the right picture shows the decidual cells induced for 6 days, Scale bar: 30μm (B) Real-time fluorescence quantitative PCR technology was used to detect the mRNA expression of decidual marker molecules IGFBP1 and PRL, GAPDH was used as internal reference. (C) RT-PCR technology was used to detect RNA expression of IGFBP1 and PRL, GAPDH was used as internal reference. The data are presented as the mean ± SD from at least three independent experiments. Statistical differences are indicated as follows: *P <0.05, ** P <0.01. (TIF) [file pgen.1010018.s003.tif]

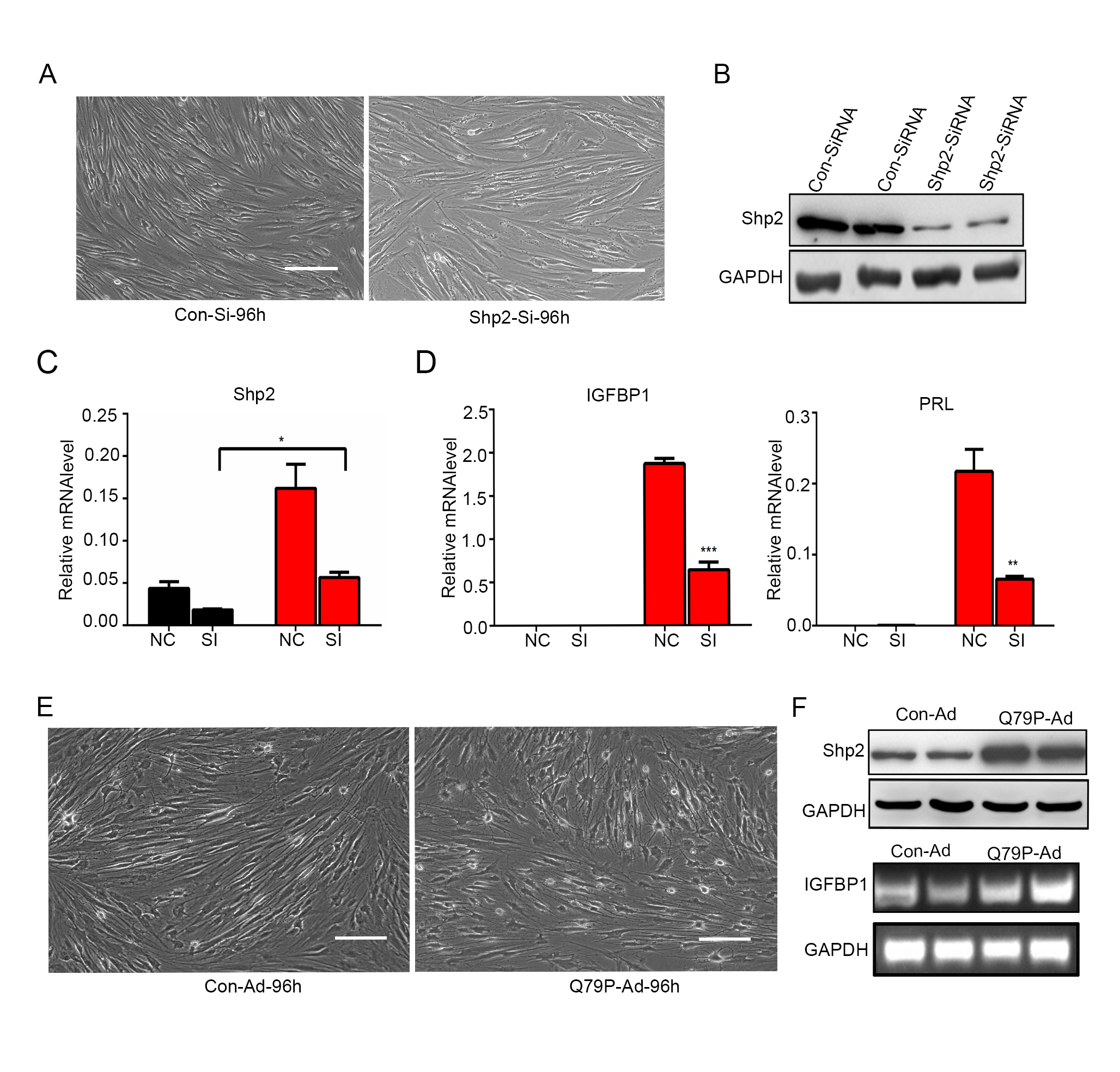

Supplement: S4 Fig — (A) Brightfield image of hESCs cultured with decidual inducer for 96h. Con-si: siRNA control, Shp2-Si: siRNA of Shp2, Scale bar: 30μm. (B) Shp2 protein in hESC transiently transfected with siRNA of Shp2 or control checked by western blotting assay. GAPDH as internal control. (C-D) The mRNA of Shp2, IGFBP1and PRL in knockdown Shp2 hESCs measured by RT-PCR. (E) Brightfield image of hESCs transiently overexpressed Shp2. Con-ad: control virus, Q79R-ad: virus expressing Shp2(Q79R), Scale bar: 30μm. (F) The top panel shows the protein expression of Shp2 in hESCs transiently overexpressed Shp2 detected by western blotting, GAPDH is the internal reference; The bottom panel shows the mRNA level of IGFBP1 in hESCs transiently overexpressed Shp2 checked by RT-PCR. GAPDH is the internal reference. The data are presented as the mean ± SD from three independent experiments. Statistical differences are indicated as follows: * P <0.5, *** <0.01. (TIF) [file pgen.1010018.s004.tif]

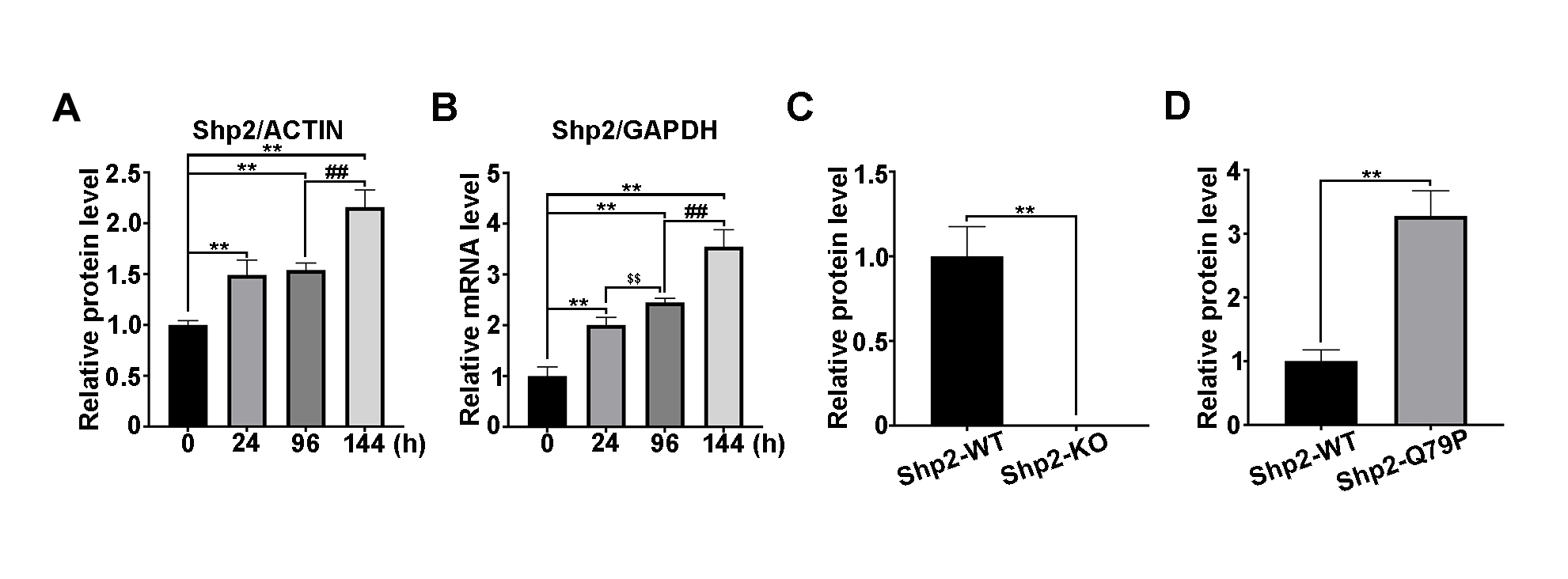

Supplement: S5 Fig — (A) Quantitative analysis of protein level of SHP2 in human endometrial stromal cells (hESC) during decidualization progress. * indicated experimental group (24h, 96h and 144h) versus control group (0h); # indicated experimental group (144h) versus experimental group (96h). (B) Quantitative analysis of SHP2 mRNA level in decidualization hESC. * indicated experimental group (24h, 96h and 144h) versus control group (0h); # indicated experimental group (144h) versus experimental group (96h); $ indicated experimental group (96h) versus experimental group (24h). (C) Quantitative analysis of protein expression of SHP2 in hESC stable knockout SHP2. (D) Quantitative analysis of protein expression of SHP2 in hESC stable overexpressed SHP2. The data are presented as the mean ± SD from three independent experiments. Statistical differences are indicated as follows: *P <0.05; **,##,$ $P <0.01. (TIF) [file pgen.1010018.s005.tif]

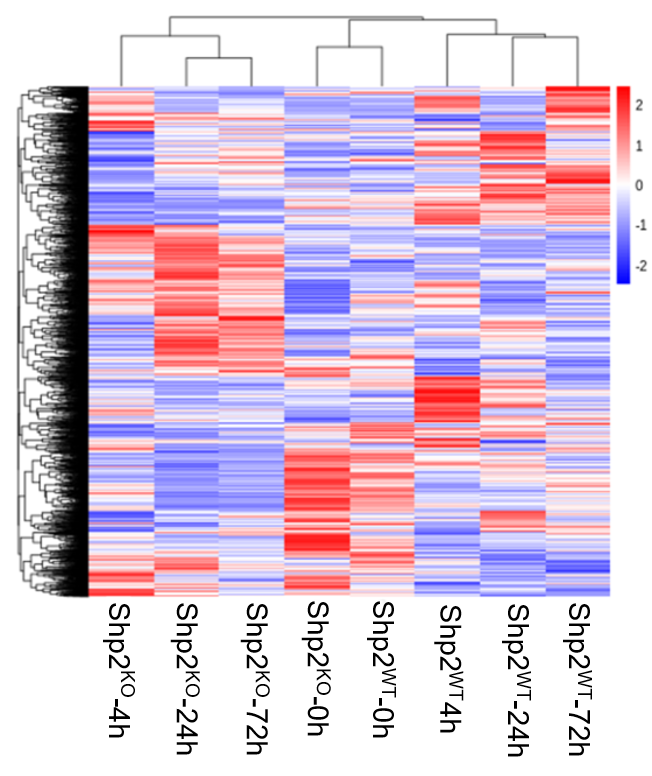

Supplement: S6 Fig — The threshold of differential genes is that any data conforms to | log2 (FoldChange) |> 0&padj <0.05. X axis is the sample cluster, and Y axis is the genes cluster. Red represents up-regulated gene transcription and blue represents down-regulated gene transcription. Color change shows the value of log2 (FoldChange). (TIF) [file pgen.1010018.s006.tif]

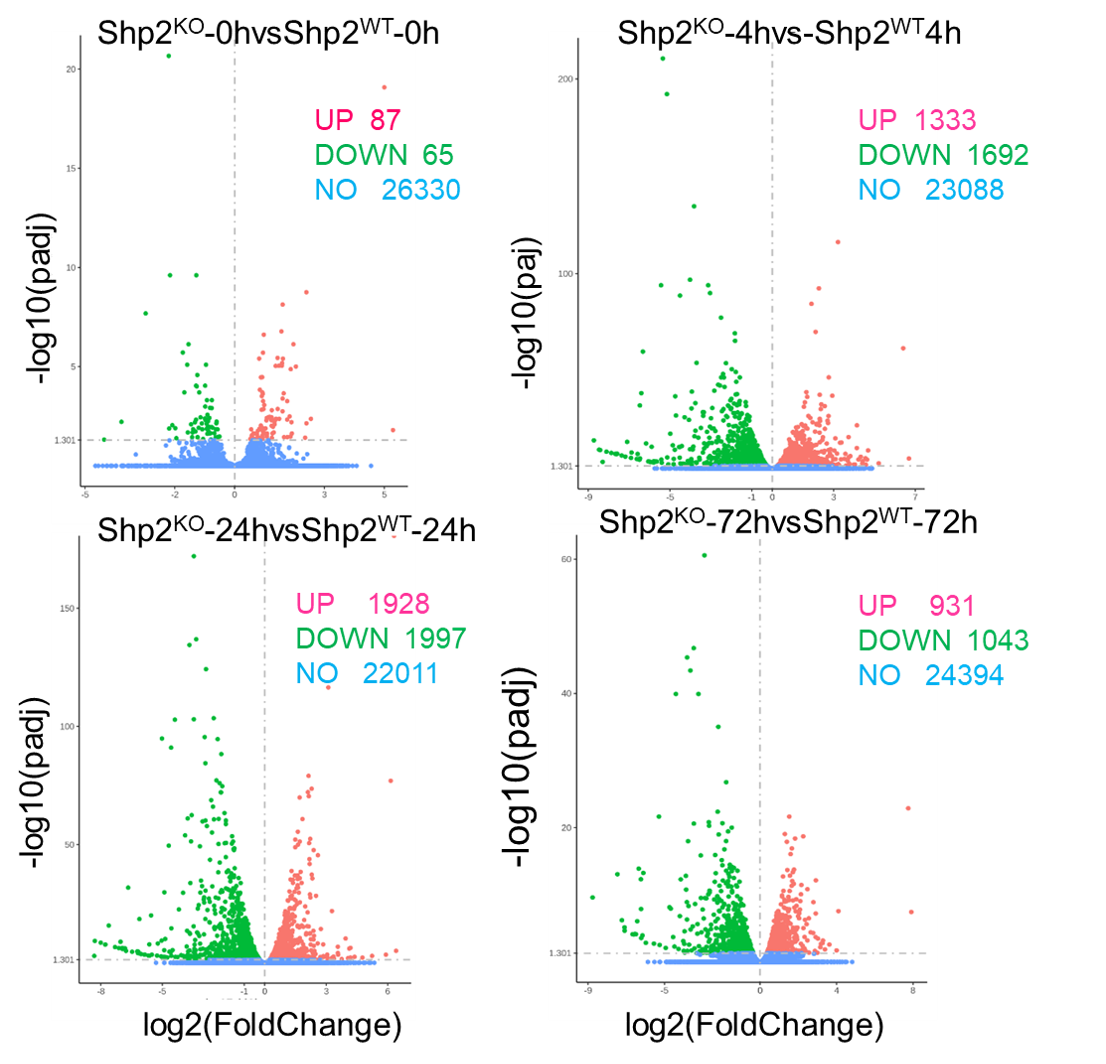

Supplement: S7 Fig — The threshold of differential genes is that any data conforms to | log2 (FoldChange) |> 0&padj <0.05. X axis is the value of log2 (FoldChange), and Y axis is -log10(padj). Red represents up-regulated gene transcription and blue represents down-regulated gene transcription. (TIF) [file pgen.1010018.s007.tif]

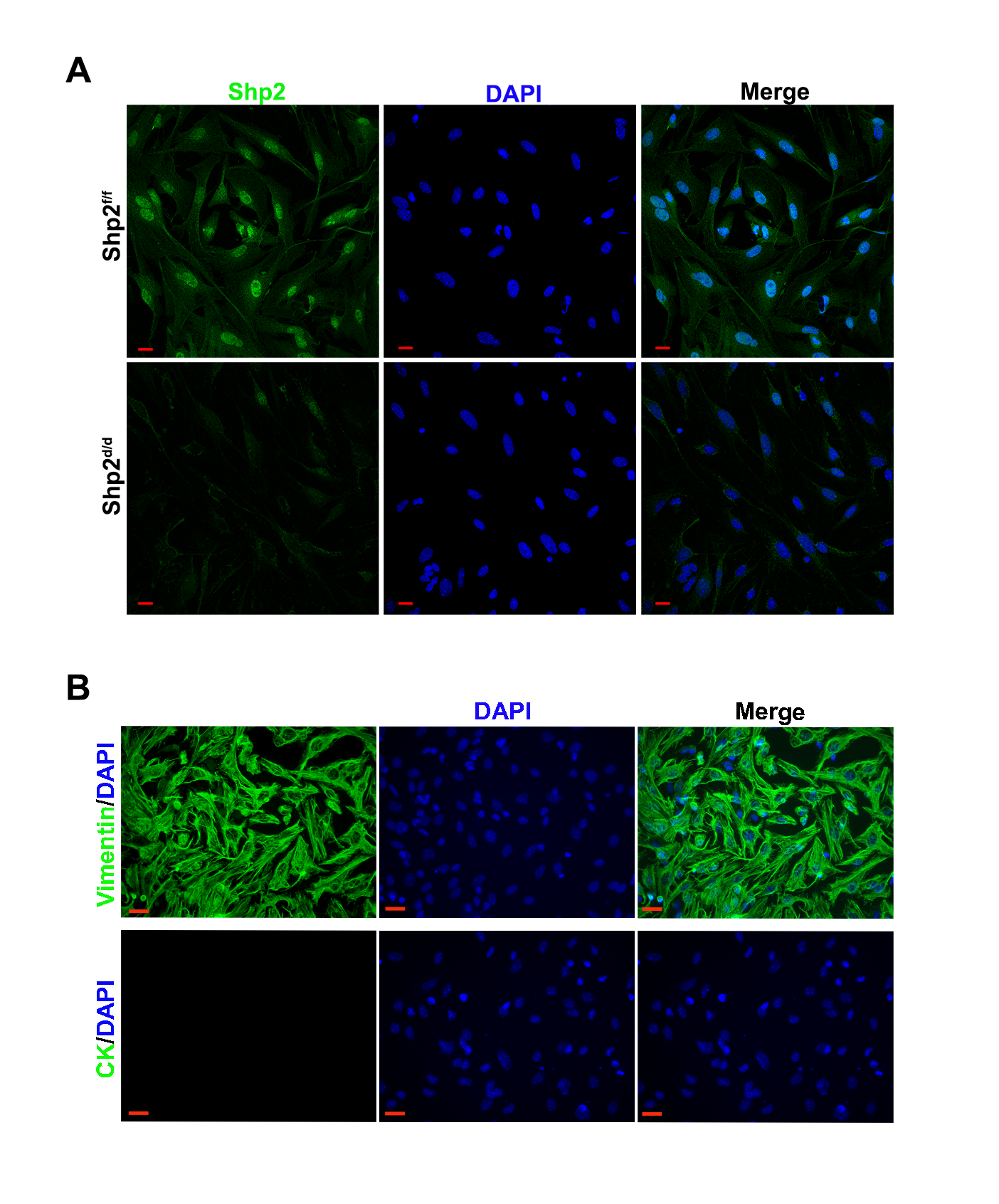

Supplement: S8 Fig — (A) The expression of Shp2 in primary stromal cells checked with immunofluorescence staining (n = 6). Green is positive color. (B)Purity assay of stromal cells with the marker protein vimentin (stromal cell marker) and cytokeratin (epithelial cell marker) labeled by immunofluorescence staining (n = 6). The cell nucleus was stained with DAPI. Scale bar: 100μm. (TIF) [file pgen.1010018.s008.tif]

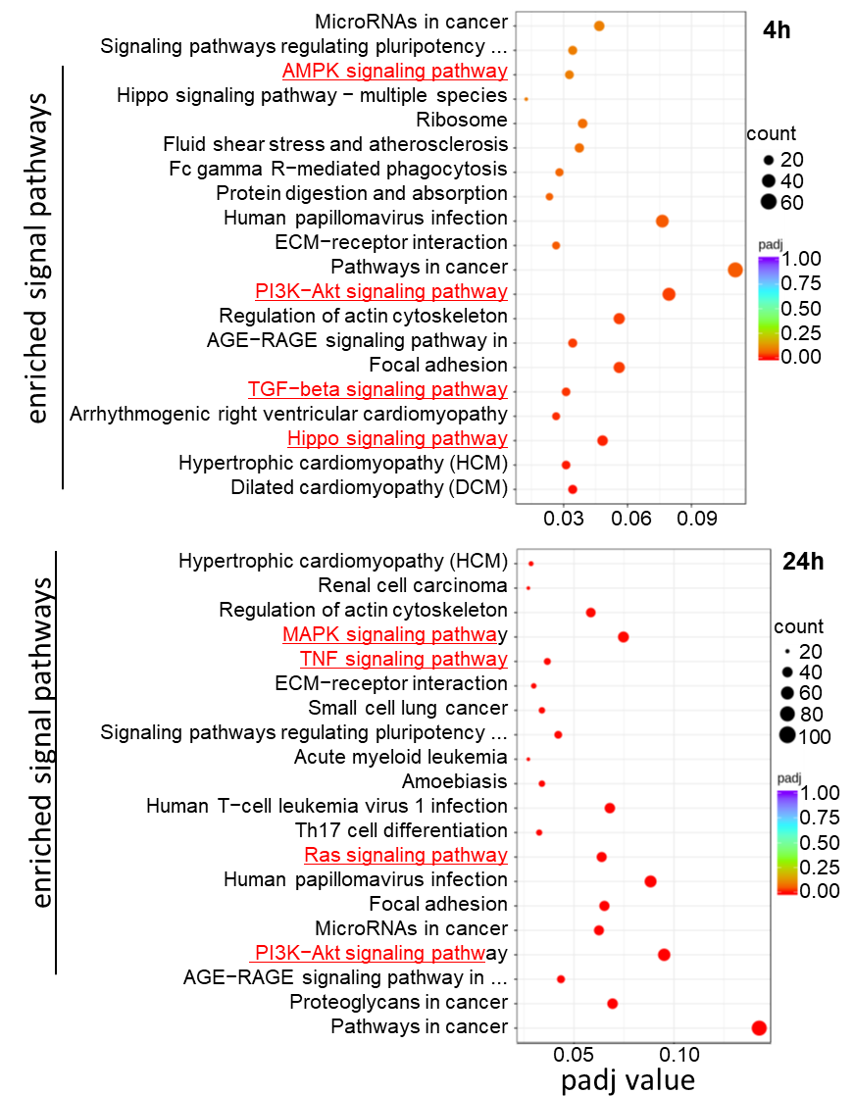

Supplement: S9 Fig — X axis is padj value and The Y-axis is enriched signal pathways. (TIF) [file pgen.1010018.s009.tif]

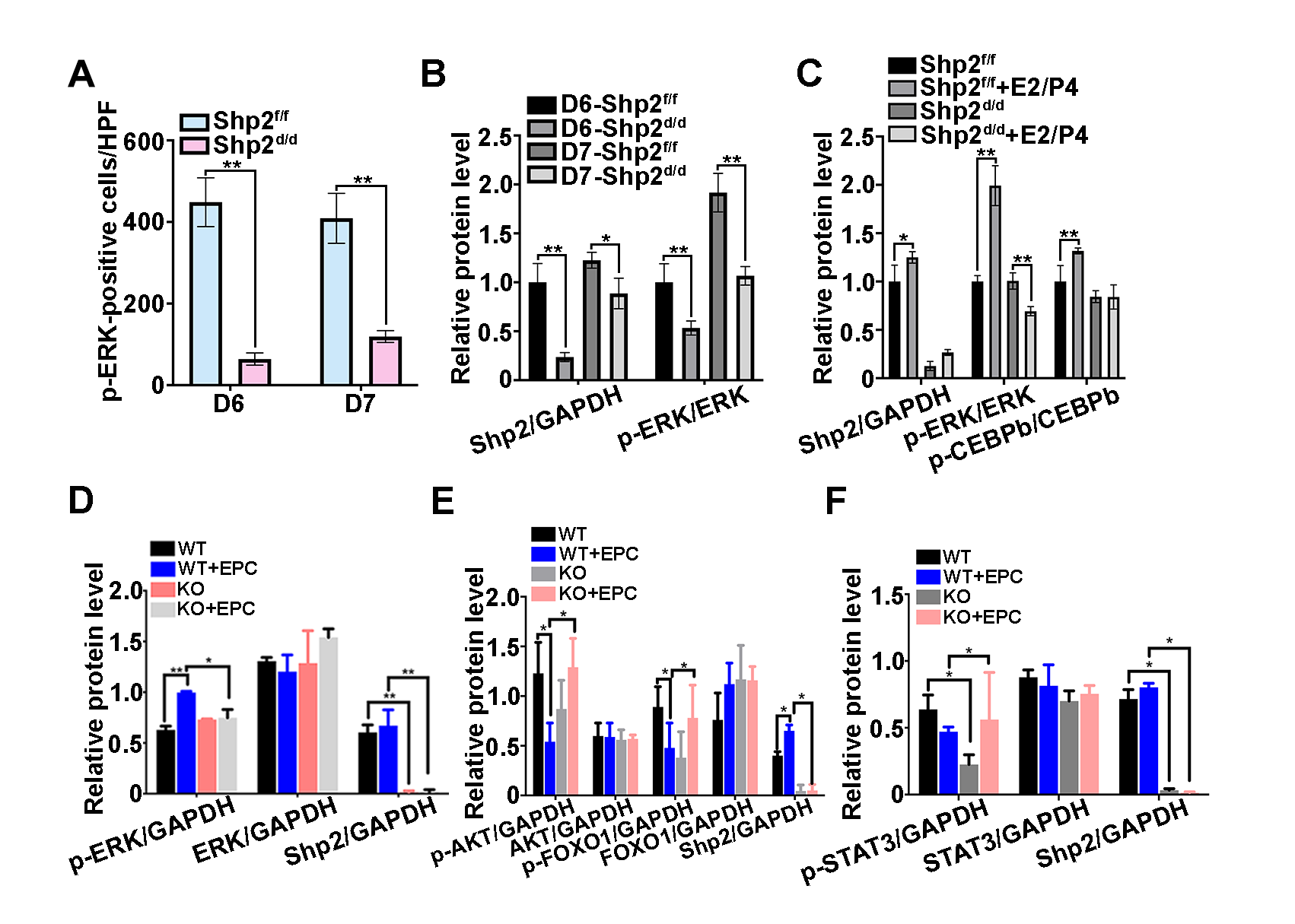

Supplement: S10 Fig — (A) Quantitative analysis of immunohistochemical phospho-Erk1/2 in the D6 and D7 IS of Shp2f/f and Shp2d/d mice. (B) Quantitative analysis of protein level of Shp2, the ratio of phospho-Erk1/2 to total Erk1/2 proteins from D6 and D7 IS in Shp2f/f and Shp2d/d mice. (C) Quantitative analysis of protein level of Shp2, the ratio of phospho-Erk1/2 to total Erk1/2 proteins, and the ration of p-C/Ebpβ to C/Ebpβ in mouse primary stromal cells treated with the decidual regimen for 24 h. (D) Quantitative analysis of protein level of Shp2, the ratio of phospho-Erk1/2 to total Erk1/2 proteins in decidual induced hESC with or without SHP2. (E) Quantitative analysis of protein level of Shp2, the phospho-AKT to total AKT proteins, and the ration of p-FOXO1 to FOXO1 in decidual hESC. (F) Quantitative analysis of protein level of Shp2, phospho-STAT3, and total STAT3 in decidual hESC. The data are presented as the mean ± SD from three independent experiments. Statistical differences are indicated as follows: *P <0.05; **P <0.01. (TIF) [file pgen.1010018.s010.tif]

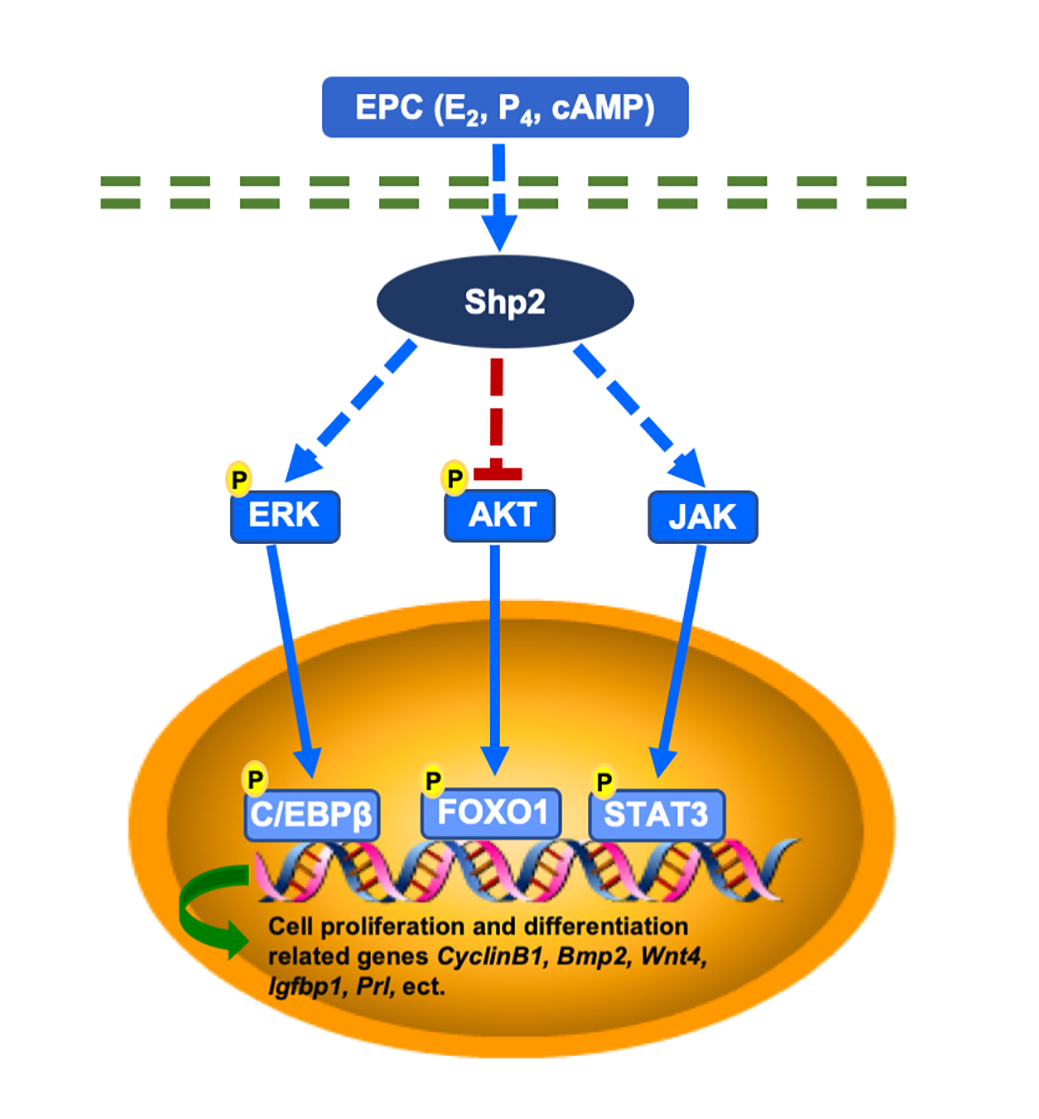

Supplement: S11 Fig — (TIF) [file pgen.1010018.s011.tif]
