# Supplementary material for: Shp2 in uterine stromal cells critically regulates on time embryo implantation and stromal decidualization by multiple pathways during early pregnancy
Source: PLoS Genet. 2022 Jan 13;18(1):e1010018. doi: 10.1371/journal.pgen.1010018 (PMC8791483; doi:10.1371/journal.pgen.1010018)
Supplement: S1 Table — (DOCX) [file pgen.1010018.s012.docx]

**S1 Table. Primary antibody used in the assay of immunohistochemistry, Immunofluorescence and western blotting.**

| **Antibody** | **Cata. No** | **Company** |
| --- | --- | --- |
| Lactoferrin | 2617720 | Millipore |
| Cytokeratin | ab53280 | Abcam |
| Vimentin | ab92547 | Abcam |
| Phospho-Histone H3 Ser10 | 9701 | Cell Signaling Technology |
| Shp2 | sc-280 | Santa Cruz |
| Ki67 | ab66155 | Abcam |
| Phospho-p44/42 MAPK (Erk1/2) | 4370 | Cell Signaling Technology |
| Cox2 | RB-9072 | Thermo Scientific |
| PR  ER  Muc1  Phospho-CEBPβ  CEBPβ  Cyclin B1  CDK1  Cyclin D3  ERK1(K-23)  Phospho-FOXO1  FOXO1  Phospho-STAT3  STAT3  Phospho-AKT  AKT  GAPDH  DAPI  β-Actin | IR068  sc-542  NB120-15481  3084  sc-150  D5C10  DCS22  D16H11  sc-94  9464s  2880  9145s  12640s  4060S  4691  60004-1-lg  H-1200-NB  66009-1-lg | DAKO  Santa Cruz  Novus Biologicals  Cell Signaling Technology  Santa Cruz  Cell Signaling Technology  Cell Signaling Technology  Cell Signaling Technology  Santa Cruz  Cell Signaling Technology  Cell Signaling Technology  Cell Signaling Technology  Cell Signaling Technology  Cell Signaling Technology  Cell Signaling Technology  Proteintech  Novus Biologicals  Proteintech |
