# Supplementary material for: Shp2 in uterine stromal cells critically regulates on time embryo implantation and stromal decidualization by multiple pathways during early pregnancy
Source: PLoS Genet. 2022 Jan 13;18(1):e1010018. doi: 10.1371/journal.pgen.1010018 (PMC8791483; doi:10.1371/journal.pgen.1010018)
Supplement: S2 Table — (DOCX) [file pgen.1010018.s013.docx]

**S2 Table. Primer sequences used for realtime PCR**

| **Gene** | **Forward** | **Reverse** |
| --- | --- | --- |
| *Muc1* | AGCCCCTATGAGGAGGTTTCG | AAGTGGTCACCACAGCTGGG |
| *Ltf* | CAGGAAAGCCCCCCTACAAA | TTCTTCACGACTGCTACCGCATA |
| *Lif* | GGCAACCTCATGAACCAGAT | TAGGCGCACATAGCTTTTCC |
| *Hand2* | AGATCAAGAAGACCGACGTGA | CTGTCCGGCCTTTGGTTTTC |
| *Areg* | CAGCGAGGATGACAAGGACCTA | GATAACGATGCCGATGCCAAT |
| *Hoxa10* | CCTAGAGATCAGCCGTAG | GACGTTGTCTGGAAGTTT |
| *Bmp2* | GAGAAAAGCGTCAAGCCAAAC | GGTGCCACGATCCAGTCATT |
| *Cx43* | GCAGACCGACGGGGTCAACG | CACCTGGTGGGGGCAGGGAT |
| *Gapdh*  *SHP2*  *IGFBP*  *PRL*  *C/EBPβ*  *IL-11Rα*  *STAT3*  *VEGFA*  *FOXO1*  *BMP2*  *WNT4*  *PGR*  *CYCLIN D3*  *GAPDH*  *Dtprp* | TGAACGGGAAGCTCACTGG  TGGTCCAGACAGAAGCACAG  TGCTGCAGAGGCAGGGAGCCC  CATCAACAGCTGCCACACTT  CAAGAAGACCGTGGACAAGC  TAGGGCATGAACTGGTCCTG  AAAGCAGCAAAGAAGGAGGC  GGCCAGCACATAGGAGAGAT  CCGAGCTGCCAAGAAGAAAG  AATGCAAGCAGGTGGGAAAG  ACAGTCGTTTGTGGATGTGC  ATGGAAGGGCAGCACAACTA  CCTGGATCGCTACCTGTCTT  GGAGCGAGATCCCTCCAAAAT  TTATGGGTGCATGGATCACTCC | TCCACCACCCTGTTGCTGTA  GGCTCTGATCTCCACTCGTC  AGGGATCCTCTTCCCATTCCA  CGTTTGGTTTGCTCCTCAAT  AGCTGCTCCACCTTCTTCTG  GTAAACCGCTGATCTGGCTG  CTGGCCGACAATACTTTCCG  TTTAACTCAAGCTGCCTCGC  ATGCACATCCCCTTCTCCAA  GCTGTGTTCATCTTGGTGCA  ACTTCTCCTTCAGTGCGTGA  AGGGCTTGGCTTTCATTTGG  GGCCAGGAAATCATGTGCAA  GGCTGTTGTCATACTTCTCATGG  CCCACGTAAGGTCATCATGGAT |
