# Supplementary material for: Shp2 in uterine stromal cells critically regulates on time embryo implantation and stromal decidualization by multiple pathways during early pregnancy
Source: PLoS Genet. 2022 Jan 13;18(1):e1010018. doi: 10.1371/journal.pgen.1010018 (PMC8791483; doi:10.1371/journal.pgen.1010018)
Supplement: Attachment — Submitted filename: Rebuttal letter_FJ-21-0328.docx [file pgen.1010018.s015.docx]

No. FJ-21-0328

Nov 09, 2021

Dear Editors and Reviewers:

Thank you for your letter and for the reviewers’ comments concerning our manuscript entitled “Shp2 in uterine stromal cells critically regulates endometrial epithelial remodeling and stromal decidualization by multiple pathways during early pregnancy”. Those comments are all valuable and very helpful for revising and improving our paper, as well as for guiding our future researches. We have studied the comments carefully, revised the manuscript accordingly and proofread the manuscript carefully to minimize typographical and grammatical errors. In addition, we listed Dr. Jianghong Cheng as a first author based on her contribution in revised manuscript and all authors agree with this change in authorship order. Revised portion are marked in red in the paper. The main corrections in the paper and the responds to the reviewer’s comments are as follows:

**Response to reviewer #1:**

**Comment 1：**The knockout efficiency of Amhr2Cre is known to be variable in different regions of the female reproductive tract (mesometrial vs. antimesometrial). Given that Shp2 is expressed throughout the stroma on day 6 of pregnancy (2017 PNAS paper), the authors should provide whole implantation site images of Shp2 IHC for control and knockout mice on day 6 in figure one.

**Answer 1:** As requested, we have provided the images of Shp2 IHC staining in whole implantation site for control and knockout mice on day 6 in the **revised figure 1A**. The staining of Shp2 in stromal cells was mostly absent in the uteri of Shp2^d/d^ mice as compared to Shp2^f/f^ mice.

**Comment 2：**In line with the previous comment, the authors should provide a lower magnification of the image presented in figure 2H to demonstrate the change in PTGS2 localization better.

**Answer 2:** Thanks for your helpful advice. To clearly demonstrate the change in PTGS2 localization, a lower magnification of the PTGS2 IHC images in control and knockout mice were provided in the revised Figure 2H. Actually, PTGS2(Cox2) correctly localized at the mesometrial pole in Shp2^f/f^ mice, while Cox2 displayed a pattern resembling the localization during the attachment in Shp2^d/d^ mice (revised Figure 2H).

**Comment 3:** Adding a schematic flow chart to Figure 6 would help the reader follow the presented results and mechanism proposed in this figure.

**Answer 3:** We are appreciated for your good suggestion. We have provided a schematic flow chart (**Supplemental Fig. 11**) to clearly illustrate the mechanism proposed in Figure 6 and to help the reader follow the presented results.

**Comment 4**
Implantation site weight is referred to in line 190 but is not shown in the figure.

**Answer 4:** We are sorry for the carelessness. Accordingly, the implantation site weight image has been added in the revised Figure 1F. The results showed that the IS weight was strikingly decreased on day 12 of pregnancy in Shp2^d/d^ mice compared with Shp2^f/f^ mice (**revised Figure 1F**).

**Comment 5**
It is important to clarify in the methods and figure legends if the hESC are primary or immortalized cells.

**Answer 5:** We are sorry for the carelessness. Actually, hESC used in this study was an immortalized cell line. We have provided the detailed information in methods and figure legends sections of the revised manuscript.

**Reviewer #2:**

**Comment 1**
The generation of the conditional knockout of Shp2 is not clearly defined and the authors need to demonstrate the other endocrine or reproductive function associated organs having a deletion of Shp2.

**Answer 1:** Thanks for your valuable advice. Amhr2 is the anti-Müllerian hormone (AMH) type II receptor (also known as Müllerian inhibiting substance type II receptor), which is expressed in the mesenchyme of fetal Müllerian ducts, the anlagen of the uterus, oviducts, cervix and upper portion of the vagina (References: Biol Reprod 2009, 81:545-552; Biol Reprod 2010, 82:422-432; PNAS 2008, 105:12469-12473). We didn’t observe any abnormal in the function of oviducts, cervix and upper portion of the vagina in Shp2^d/d^ mice. We have added this information in the revised text.

**Comment 2**

Line no. 162-165 and Fig S.1. The densitometric analysis, figure 1 A-S, is unclear.

**Answer 2:** We are sorry for the fuzzy description in the densitometric analysis of Figure S1. Actually, the histogram in the right panel of figure S1B was the relative quantitative analysis of protein levels of Shp2 from D4 to D8. We have rephrased the Y-axis of this histogram (relative protein expression of Shp2—relative to GAPDH).

**Comment 3**

Line no. 165-167. To know the differential expression of Shp2, a large area or rather full uterine tissue image is required.
**Answer 3:** We are appreciated for your insightful advice. We have provided the Shp2 IHC results using full uterine tissues on day 4 to day 7 in the revised figure S1C (**Figure S1C**, the lower images).

**Comment 4**

What is the reason for the authors to study the expression level of Shp2 at Day12 rather than Day6?

**Answer 4:** I apologize for confusing you with our data. We analyzed the expression level of Shp2 on Day 6 which were presented in Figure 1B-1C and supplementary figure 1.

**Comment 5**

How was the ovulation/ ovulated eggs as earlier images, it's not clear whether they have any difference in wild type than the wild type?

**Answer 5:** We are sorry for the ambiguous description. We could not compare the ovulation/ovulated eggs in wild-type mice of figure S2A to that in figure S2B. Actually, we mainly observed the morphology of ovary and follicular development in 8-week-old mature shp2^f/f^ and shp2^d/d^ female mice in Figure S2A (**Fig. S2A**). However, we employed the super-ovulation by injecting PMSG and hCG into 3-week-old immature female mice to observe their response ability to gonadotropin in Figure S2B (**Fig. S2B**). We have rephrased these sentences in the revised manuscript.

**Comment 6**

The serum levels of P2&E2 during the pregnancy failed either normal P2 &E2 or Shp2 downstream signaling.
**Answer 6:** Thank you for your valuable comment. Serum P4&E2 were mainly produced by ovarian cells of during the pregnancy. Serum levels of P4&E2 on day 4 of pregnancy was very important for the normal embryo implantation and decidualization in mice. In the present study, Shp2 deletion did not affect serum levels of P4&E2 on day 4 of pregnancy (**Fig. S2C-2D**). Additionally, the expression of PR and ER were also not affected by Shp2 deletion. However, Shp2 removal might regulate the alterations of P4&E2-related downstream signaling pathway as compared to that in wild-type mice.

The downstream signaling of Shp2 in this study mainly focused on the changes of intrauterine signals. Although serum P4&E2 level had no differences between the two types of mice, other downstream signaling pathway mediated by intrauterine P4&E2 might be regulated by Shp2 deletion. In addition to that, the changes of P4&E2-related signaling pathway in Shp2 knockout mice possibly caused by other molecules which could also initiate these signaling pathways.

**Comment 7**

The six months’pregnancy is unclear and how many reproductive cycles have been covered /achieved, a detail of it is required.

**Answer 7:** As requested, we have provided the detailed information about the six months’pregnancy assay. In brief, Shp2^f/f^ and Shp2^d/d^ females were placed in a cage 1:1 with WT males of proven fertility for consecutive 6 months. During the six months’pregnancy assay, the mice were observed every 3 days and the number of pups per litter were recorded. No differences in reproductive cycles were found between Shp2^f/f^ and Shp2^d/d^ female mice. At the end of experiment, the total number of litters were calculated. We have added these informations into the result and Method sections in the revised manuscript.

**Comment 8**

Whether the eggs of both wild type and null have any visual or other difference is not clear.

**Answer 8:** There were no difference in morphology and number of the super-ovulated eggs between Shp2^f/f^ and Shp2^d/d^ groups. Unfortunately, we did not take pictures of the super-ovulated eggs after the super-ovulation assay. We are sorry for not providing the photos of super-ovulated eggs.

**Comment 9**

The serum level of the P4 and E2 during the pregnancy period is suggested to be examined to know the hormonal and Shp2 cross-talk if any.
**Answer 9:** Originally, we focused on the role of Shp2 on embryo implantation. Due to the critical role of serum levels of P4&E2 on day 4 of pregnancy for normal embryo implantation and decidualization in mice, we only detected the secretion of P4&E2 on day 4 of pregnancy in both types of mice. Serum samples at other time points were not collected and thus serum levels of P4 and E2 at these time points were not monitored. We are sorry for the negative response.

**Comment 10**

Line no. 222. It must be reduced proliferation, rather than attenuation.
**Answer 10:** We are sorry for the careless. We have rephrased this sentence according to your comment.

**Comment 11**

Line nos. 225-227. Please confirm by epithelial biomarkers to claim that Epithelial cells are retained & assess from different replicates.
**Answer 11:** We appreciate your valuable suggestion. Accumulating evidence has confirmed that cytokeratin (CK) is a potent epithelial cell marker (Shear et al., Oral Oncol. 2002 Jul;38(5):407-15; Rickmeyer et al., Histol Histopathol. 2019 Jun;34(6):683-696). Therefore, immunostaining of epithelial cell markers CK can be used to determine the state of luminal epithelial layer. We have made this clear in the revised manuscript.

**Comment 12**

Line no.232. Why cox2 is having studied now any better rationale for that?
**Answer 12:** This is our negligence. In normal physiological conditions of mouse implantation, dynamic localization of Cox2 precisely correlates with different phases of the implantation process, and the location of Cox2 shifts from the stroma surrounding the embryos to the mesometrial pole between D5-D8. The abnormal localization of Cox2 in peri-implantation uterus may indicate the deferred embryo attachment. In the present study, Cox2 correctly localized at the mesometrial pole in Shp2^f/f^ mice on D6, (**Fig. 2H,** arrowhead indicated in the top two images), in contrast to that in Shp2^d/d^ mice, which displayed a pattern resembling the localization during attachment (**Fig. 2H**, arrowhead indicated in the below two images). Thus, the implantation process was deferred in Shp2^d/d^ mice compared to that in Shp2^f/f^ mice. According to your comments, we have added the detailed information in the revised manuscript.

**Comment 13**

Line nos. 263-266. The quantitative analysis of Dtprp by staining intensity analysis as well as immunoblot / PCR is required to validate the given author's statement.

**Answer 13:** As per your comments, we have performed the quantitative analysis of Dtprp by staining intensity analysis of figure 3D. The quantitative analysis result was presented in figure 3E (**revised Fig. 3E**). Additionally, qRT-PCR assay was also used to verify the findings. As shown in Figure 3F, transcriptional level of *Dtprp* sharply decreased in Shp2^d/d^ uteri on day 8 of pregnancy as compared to that in Shp2^f/f^ mice (revised **Fig. 3F**). The primer sequences of *Dtprp* used for real-time PCR were added in the **Supplementary Table 2**.

**Comment 14**

Line 261. The statement about the “defective differentiation of uterine stromal cells” is inappropriate as the authors did not assess the differentiation process either in vitro or in vivo.
**Answer 14:** It’s a typo. Accordingly, we have rephrased this sentence into “defective decidualization of uterine stroma cells” which was highlighted in red in the revised manuscript.

**Comment 15**

Figure 4 & Lines 303-303. The Shp2 protein &mRNA levels were not quantified: hence, quantitative/ relative expression in fig 4. A, B, C, F, S, and D, can not be stated. Likewise in figure- 6 B, C, E, D, and G.
**Answer 15:** As per your advice, we have provided the quantitative analysis about the Western blotting assay mentioned by you. The quantitative analysis results were presented in the corresponding supplementary figures.

**Comment 16**

Figure 6. The fluorescence intensity value calculation is not done and visually the expression can not be appropriate to show semi-quantitative/comparative.
**Answer:** According to your comments, the quantitative analysis of P-ERK-positive cells in figure 6A has been provided in the supplementary figures (**Supplemental Fig. 10A**).

**Comment 17**

Line386. The statement has to be rephrased according to the localization pattern as it happened to be restricted to the epithelial region around the trophoblast site.

**Answer:** We are sorry for the vague description. Actually, p-ERK mainly expressed in uterine stromal cells in both Shp2^f/f^ mice and Shp2^d/d^ mice on days 6-7 of pregnancy. However, deletion of Shp2 drastically inactivated Erk1/2 activity by contrast to the wild-type group. We have rephrased the sentence in the revised manuscript which was highlighted in red*.*

**Comment 18**

18. Figure 6 B, C, and D. Please explain the double band of ERK probed blot.

**Answer:** We are sorry for our incomplete description in antibody for ERK. In the present study, Phospho-p44/42 MAPK (Erk1/2) (Cat. 4370, CST) and ERK1(K-23 (sc-94, Santa Cruz) antibodies could detect double band (p44/42). We have provided full information of ERK antibody in revised Supplemental table 1.

**Comment 19**

Figure 6F. FOXO1 double band and followed the trend to be explained.
**Answer 19:** In the present study, FoxO1 (Cat. 2880, CST) and Phospho-FoxO1 (Ser256) (Cat. 9461, CST) antibodies could detect double band (FoxO1 and FoxO3a) (see detail in the official website of Cell Signaling Technology. We are sorry for our error information of FoxO1 antibody in Supplemental Table 1. We have corrected it in revised manuscript.

We are also sorry for our mistake in description of FOXO1 results. We have redescribed FOXO1 and P-FOXO1 trends in the revision.

**Comment 20**

Figure 6. The ERK, etc. expression or phosphorylation can also be linked to decidualization. However, the author's statement about the Shp2 dependent ERK signaling needs to be experimentally explained using normal stromal cells post Shp2 gain and loss functions then the Shp2 dependent ERK regulation can be stated.
**Answer 20:** We are appreciated for you insightful advice. Shp2 dependent ERK signaling has been proved in the isolated primary stromal cells from Shp2^f/f^ mice and Shp2^d/d^ mice in the present study. As you said, we also have confirmed this finding using the immortalized human endometrial stromal cells that were knocked out of Shp2 or not (**Fig. 6D**). For the statement about the Shp2 dependent ERK signaling, we have thoroughly corrected the inappropriate descriptions in the revised whole text.

**Comment 21**

Line 434. The Shp2 is not only ablated from the stromal cells as discussed.
**Answer 21:** It’s a typo. We have corrected the wrong description in the “Discussion” section of revised manuscript.

**Comment 22**

A language check is suggested.

**Answer 22:** As requested, we have thoroughly corrected the whole text and the manuscript has been polished by Nature Publishing Group Language Editing again. The certification (No: 349D-DD0E-1B8E-BDD6-5C3P) has been uploaded through the submission system. (secure.authorservices.springernature.com/certificate/verify).
