# Supplementary material for: Shp2 in uterine stromal cells critically regulates on time embryo implantation and stromal decidualization by multiple pathways during early pregnancy
Source: PLoS Genet. 2022 Jan 13;18(1):e1010018. doi: 10.1371/journal.pgen.1010018 (PMC8791483; doi:10.1371/journal.pgen.1010018)
Supplement: Attachment — Submitted filename: Rebuttal letter_PGENETICS-D-21-01259R1.docx [file pgen.1010018.s016.docx]

PGENETICS-D-21-01259R1

Dec 20, 2021

Dear Editors and Reviewers:

Thanks again for your letter and for the reviewers’ comments concerning our manuscript. Following those valuable and helpful advice, we have revised the manuscript and proofread the manuscript carefully. Revised portion are marked in red in the paper. The main corrections in the paper and the responds to the reviewer’s comments are as follows:

**Response to reviewer #1:**

**Comment 1**

The authors have adequately addressed my previous comments and the manuscript is suitable for publication.

**Answer 1:** Thanks again for your efforts onto our manuscript.

**Response to reviewer #2:**

**Comment 1**

The present Manuscript seems to be an extension of the already published article ‘Nuclear Shp2 directs normal embryo implantation via facilitating the ERα tyrosine phosphorylation by the Src kinase. From the same groups such as expression analysis at d4, and 5, the impact of Shp2 deletion on the embryo implantation, proliferation studies, ovulation, litter size, hormones levels, mucin expression level, etc. seemed to be overlapping, please look at these point very carefully.

**Answer 1:** Thank you for your valuable suggestion. Actually, a mouse model harboring a uterine-specific deletion of Shp2 (*Shp2^d/d^*) was generated by crossing Shp2-loxp mice (Shp2^f/f^) with PR-Cre mice (*Pgr*^Cre/+^) in the previous study (*Nuclear Shp2 directs normal embryo implantation via facilitating the ERα tyrosine phosphorylation by the Src kinase*). Shp2 was efficiently deleted in PR-expressing uterine cells of Shp2^d/d^ females. However, in the present study, we crossed Shp2^f/f^ mice with Amhr2-Cre mice, which (*Amhr2-cre*) are widely used to delete target genes in the stroma and myometrium but not in uterine epithelial cells Therefore, shp2-deleted cell types between the two papers were different. This study is aimed to further identify the cell type-specific function of Shp2 in the uterus.

**Comment 2**

The title is vague as per the presented data set and there are no remodeling studies neither the epithelial and epithelial cross-talk studies. Hence, the title needs to reflect the generated data and not be ambiguous.

**Answer 2:** Thanks for your valuable advice. We have rewritten the title into “Shp2 in uterine stromal cells critically regulates *embryo implantation* and stromal decidualization by multiple pathways during early pregnancy”.

**Comment 3**

The detail of the generation of the conditional knockout of Shp2 is not explained in the main manuscript text.

**Answer 3:** We are sorry for confusing you with our description. Actually, in the last paragraph of “Introduction” section, we have mentioned the detail of the generation of the conditional knockout of Shp2. According to your comments, we also described the generation methods of conditional knockout of Shp2 in the second paragraph of results section, the first subheading.

**Comment 4**

Lines 85-86 are overstated about the treatment of pregnancy failure. If that is so then the authors can show the rescue experiment for Shp2 where the embryo implantation is studied in presence of recombinant shp2 supplementation in the shp2 -/- animals. To prove further, the authors can generate the pregnancy failure mouse models and see the role of shp2.

**Answer 4**: We are appreciated for your helpful advice. We have rewritten this sentence into “our discovery possibly provides a novel key regulator of embryo implantation and novel therapeutic target for pregnancy failure”.

**Comment 5**

Lines 206-208. Figures-1 C. PCR, Quantitation is necessary to know the relative change rather than just relying on the visual impression.

**Answer 5**: As per your comments, quantitative analysis of PCR assay in figure 1C has been performed, and the quantitative results have been presented in the **revised Figure 1.**

**Comment 6**

Line 209. There is no supporting data that AMHR-2 expression cells have SHP2 deletion.

**Answer 6**: Accumulating evidence have confirmed that AMHR2-cre mice is a classical tool mouse to delete target genes in the stroma and myometrium but not in uterine epithelial cells (Jamin et al., Nat Genet 32:408-410, 2002; Tanaka et al., Endocrinology 153: 404-416, 2012; Hernandez Gifford et al., Biol Reprod. 2009, 80(6):1282-92) which has been widely recognized. In the present study, we did not perform IHC staining using AMHR2 antibody. However, the data in figure 1A clearly suggested that Shp2 was obviously deleted in uterine stroma cells. We are sorry for the negative response.

**Comment 7**

Lines 212-213. The rationale to observe every 3 days is not clear as the gestation period is 21 and the estrous cycle s 4-5 days. How much gestation/pregnancy is seen during 6 months study? Over the 06 months, each pregnancy outcome is to be analyzed separately rather than cumulative.

**Answer 7**: We are sorry for the careless. It’s a typo. Actually, the mice were observed every day after pregnancy (significant signs of baby bulge) during the six-month pregnancy assay. 4-5 pregnancies were seen during 6 months study. We have corrected this error and added the data about reproductive cycles in the **revised figure 1E**. For the analysis of pregnancy outcome, cumulative analysis is the most common method for the subfertile mice. The analysis of each pregnancy outcome is often used to assess the infertile mice. Thus, we chose the cumulative analysis method in this study.

**Comment 8**

Lines 221-222. There is a lack of supporting data.

**Answer 8**: We are sorry for this carelessness. We have added the data in the **revised figure 1E** according to your comments.

**Comment 9**

Fig. S9. It can be explained in detail as there is no caption for the x and y-axis.

**Answer 9**: As requested, we have added the caption for the x and y-axis of Fig. S9. The Y-axis is enriched signal pathways and X axis is padj value.

**Comment 10**

Lines 232-233. The ovulation does not differ between the +/+ and -/- genotypes; hence, the statement might change accordingly.

**Answer 10**: As per your comments, we have rephrased this sentence in the revised manuscript which were highlighted in red.

**Comment 11**

Lines 234-236. The serum levels E2 and P4 and not largely affected and there is no statistical significance, which can confirm the same.

**Answer 11**: We are sorry for the ambiguous description. There was indeed no difference in circulating levels of progesterone and 17β-estradiol (E2) between Shp2^f/f^ mice and Shp2^d/d^ and. We have rephrased this description according to your comments.

**Comment 12**

Line 232. The ‘02:00’ units are to be clarified.

**Answer 12**: Thank you for you suggestion. The wording “02:00” indicated “2 o'clock (02:00) in the morning on day 5 of pregnancy”. We have added the detailed information in the revised manuscript.

**Comment 13**

Line 246. What is the status of the null mouse embryos on day5, 1000 h?

**Answer 13**: Embryos had implanted in Shp2^d/d^ uteri at 10:00 on day 5 of pregnancy by Chicago blue staining assay (**Fig. 2C**). However, the blue bands in Shp2^d/d^ uteri were not as obvious as those in the Shp2^f/f^ group (**Fig. 2C**), indicating delayed implantation in Shp2^d/d^ mice.

**Comment 14**

Line 251-254. It would ideally be good to study the cell proliferation markers in the null mouse.

**Answer 14**: Thank you for your helpful advice. Ki67 was a classic cell proliferation marker. We have performed the IHC staining using Ki67 antibody in the uterine of Shp2^f/f^ mice and Shp2^d/d^ mice. Ki67-positive cells were not observed in the in the uterine LEs of Shp2^f/f^ mice on D4 of pregnancy, while Ki67-positive cells were exhibited in some uterine LEs of Shp2^d/d^ mice.

**Comment 15**

Fig. 2F. How much area of tissue has been used to calculate the % change in Ki67 staining?

**Answer 15**: Actually, six HPFs per tissue were used to calculate the positive rate of Ki67. We have added the detailed information in legends of Fig. 2F.

**Comment 16**

Lines 296-297. The data to support the area of IS is not presented to deduce any information.

**Answer 16**: We are sorry for confusing you with our description. Actually, in figure 3A, the arrowheads indicated the implanted embryos in both Shp2^f/f^ mice and Shp2^d/d^ mice. The sizes of the entire implantation sites (ISs) on D6-8 of pregnancy in Shp2^f/f^ mice and Shp2^d/d^ mice were monitored by using HE staining (Fig. 3A). The separate implantation site (IS) was dissected from the uterus and weighted to assess the weight of each IS on D6-8 of pregnancy in Shp2^f/f^ mice and Shp2^d/d^ mice. Both methods were used to prove the defective decidualization in Shp2^d/d^ mice.

**Comment 17**

In each of the experimental figure /result sections, mentioning the replicate's number will be more clear to the reader.

**Answer 17**: Thank you for your valuable advice. Actually, the data are obtained from at least 6 mice (6-10) in each group or three independent experiments. As requested, we have provided this replication information in results and figure legends.

**Comment 18**

Line 362. Please look at the ‘padj’.

**Answer 18**: The wording “padj” indicated “p-adjusted p value (padj)”. We have provided the full name of “padj”.

**Comment 19**

Figure-S6. It needs a better presentation to be clear to the reader rather than just only a heat map.

**Answer 19**: Thank you for your good suggestion. We have provided more information in line 365-374 in our revised manuscription.

**Comment 20**

Likewise, figure-7 needs a clear presentation of the genes found.

**Answer 20**: Thank you for valuable advice. We have descripted the altered genes again in line 377-378 and figures legend in our revised manuscription.

**Comment 21**

Line 520 , 581, 1108, etc. Please correct the typo error.

**Answer 21**: According to your comments, we have carefully checked the whole text and corrected the typo errors in the manuscript.

Thank you for your time and consideration. We look forward to hearing from you.

Sincerely,

zhongxian

Zhongxian Lu

Professor,

School of Pharmaceutical Sciences

Zhuangjin Hall, Room 319, Xiamen University

Xiamen, Fujian 361102, China

phone& fax: 86-592-2187227

Email: zhongxian@xmu.edu.cn
